# The hearing function of sound engineers: A hearing conservation perspective

**DOI:** 10.4102/sajcd.v67i1.638

**Published:** 2020-04-28

**Authors:** Liepollo Ntlhakana, Angie Heliopoulos

**Affiliations:** 1 Department of Speech Pathology and Audiology, University of the Witwatersrand, Johannesburg, South Africa

**Keywords:** noise-induced hearing loss, hearing conservation programmes, hearing protection devices, sound engineers, decibels

## Abstract

**Background:**

Occupational activities performed by sound engineers are associated with hearing loss. However, there is a dearth of research on the hearing functions and the related hearing loss for sound engineers.

**Objectives:**

To determine the hearing function and early effects of noise on the hearing ability of sound engineers, and to establish whether there are hearing conservation programmes (HCPs) available for sound engineer participants in Johannesburg.

**Methods:**

A quantitative cross-sectional design was used. Eight sound engineers from the Academy of Sound Engineering (ASE) participated in the study. The following data were collected from the participants: case history data, ear-related symptoms and hearing conservation data. Hearing screening followed by full diagnostic audiological assessments was conducted for each participant. Descriptive statistics and one-sample *t*-test were used to analyse the data (confidence interval [CI] = 95%).

**Results:**

Participants reported tinnitus and aural fullness as common complaints. Only one participant had a unilateral impacted cerumen. All the participants presented with hearing within normal limits bilaterally, with a percentage loss of hearing (PLH) of 1.1% for all the participants. However, five participants presented with a notched configuration that was ≥ 10 decibel (dB), in the high frequency region at 3000 Hz and 6000 Hz bilaterally. One-sample *t*-test (*p* = 0.001) inferred that at a mean age of 27.6 years (standard deviation [SD] = 3.85), a notch at 3000 Hz and 6000 Hz was associated with an early sign of a hearing loss for the study participants. An HCP was not in place at the study site.

**Conclusion:**

The study indicated a younger age as an associated early sign of noise-induced hearing loss (NIHL) for the study participants, and that audiologists’ clinical practice needs to explore HCP strategies specific for the sound engineers in order to prevent hearing loss.

## Introduction

Hearing loss because of a prolonged exposure to loud music emulates signs of noise-induced hearing loss (NIHL) in the organ of Corti (Rabinowitz, 2000). Noise-induced hearing loss can be defined as hearing loss caused by continuous or intermittent noise exposure, which may develop slowly over several years (Kirchner et al., 2012). This type of hearing loss is sensori-neural in nature and is preventable (Rabinowitz, 2000; World Health Organization [WHO], 2010). On a plotted audiogram, NIHL is typically recognised with a ‘notched’ configuration usually seen around 4000 hertz (Hz) in the region of 3000 Hz – 6000 Hz (Feuerstein, 2009; Rabinowitz, 2000). In South Africa, hearing conservation policies exist in order to reduce noise exposure levels to below 85 decibel A (dBA) averaged over an 8h period and prevent NIHL (South African National Standards [SANS], 2013). Noise-induced hearing loss is an irreversible and a permanent type of hearing loss, which is also associated with tinnitus and aural fullness. Because of the nature of NIHL, affected individuals are unaware of the deterioration in their hearing acuity and only realise the problem when they have a hearing loss (Cruz, 2005).

The World Health Organization has provided a list of professionals at risk of developing NIHL, and professionals in the music industry appear in this list as well. Sound engineers’ professional work relies strictly on their hearing acuity as they integrate separate sounds to formulate one sound (Sokanu, 2018). They are involved in mixing, producing, recording and adjusting sounds together for television (TV) advertisements; they also work with radio station transmissions, musical groups in recording studios and live events. Some of the tools that sound engineers work with include microphones, speakers, recorders and amplifiers (Hynes, 2007). The work environment and tools used by sound engineers emit high levels of noise, increasing susceptibility to hearing loss (CareerExplorer, 2018).

Early identification and prevention of hearing loss for sound engineers is important (Marshall, 2018). Therefore, a need to monitor their hearing function within a hearing conservation programme (HCP) is imperative. In a study from 1997 to 2000 conducted by Cruz and Potthoff (2005), the following audio industry professionals – the Audio Engineering Society, the National Sound Contractors’ Association, the National Association of Music Merchandisers and Lighting Design International – all reported hearing within limits following hearing screening assessment. Although the participants presented with hearing screening results within normal limits as indicated on the pure-tone audiogram, a notch in the 3000 Hz – 6000 Hz frequency range was evident, which indicated an early sign of NIHL (Rabinowitz, 2000). Thus, the researchers highlighted the importance of continuous hearing screening for the prevention of NIHL.

For a profession that relies heavily on the use of ears, there is no mention of HCPs integrated in the training of sound engineers (Academy of Sound Engineering, 2019). This results in professionals continuously displaying early signs of NIHL. Furthermore, research on the hearing functions and effects of noise exposure on audio engineering professionals remains scarce (Bulla, 2003).

In South Africa, there are several studies that exist on hearing and the harmful effects of working in a noisy environment (Chamber of Mines, 2016; De Jager, 2017; Edwards, Dekker, Franz, Van Dyk, & Banyini, 2011; Edwards, Malanzi, Khoza, & Zungu, 2015). However, the focus has been placed on the regulated mining and construction industries (Balfour-Kaipa, 2014; Chung, 2007). This could be influenced by factors such as contribution to the country’s workforce and the financial contributions made by these industries towards the country’s gross domestic product (GDP). Sound engineering does not feature on the GDP list provided by the Statistics South Africa reports; yet risks related to sound engineers’ occupation need to be monitored and addressed. There are audiological implications for this professional group; therefore, HCPs guided by audiologists need to be considered for purposes of early identification and monitoring of NIHL together with prevention.

## Methods

### Aim

The purpose of this study was to determine the hearing function of sound engineers and to establish if HCPs were in place at the sound engineers’ academy in Johannesburg, South Africa.

### Objectives

The objectives of this article were: (1) to determine the sound engineers’ hearing status and (2) to establish an HCP followed by the sound engineers’ academy.

### Research design

A quantitative, cross-sectional research design was used for this study. The researchers conducted audiometry assessments and established if the study site had an HCP available in place. Quantitative data were obtained from the participants’ audiometry results. In order to address the study’s aim, all the data obtained were analysed quantitatively.

### Study sample

A non-probability purposive sampling method was used for the selection of participants. The study sample included eight sound engineering lecturers employed at the Academy of Sound Engineering (ASE) in Johannesburg. The mean age of the participants was 28 years (standard deviation [SD] = 3.85 years), with only one participant above the age of 30 years. Six were white participants and seven were male participants. The recorded years of experience within the sample ranged between 3 and 16 years, with the majority (*n* = 7) of participants having worked for less than 10 years. The mean duration of noise exposure per day (in hours) was 6 h.

Table 1 shows the participants’ demographic information. The sample had only one female participant.

**TABLE 1:** Demographics of the study participants (*N* = 8).

Item	Categories	*n*
Age (years)	21–25	3
	26–30	4
	31–35	1
Race	Black African people	1
	Mixed race people	1
	White people	6
Gender	Female	1
	Male	7
Years of experience	1–5	3
	6–10	4
	11–15	0
	16–20	1
Education level	Tertiary	8

### Data collection

Data collected for case history information were based on a self-developed and administered questionnaire. Questions included signs and symptoms of hearing loss, medical and family history, occupational and recreational history. Additional data collected included hearing screening and diagnostic audiology assessment results. The audiometric protocol included otoscopic examination, immittance audiometry, pure-tone hearing screening and distortion product oto-acoustic emissions (DPOAEs). Diagnostic audiometry included additional tests for speech audiometry. Hearing screening and diagnostic audiometry were conducted 1 week apart from one another to resolve existing middle ear pathologies. Assessment tools were calibrated and calibration certificates were valid at the time of data collection.

#### Data collection procedures and tools

Testing for hearing screening and diagnostic audiometry was performed at the university audiology clinic. Results obtained from all assessment tests were interpreted by the researchers in order to confirm the participants’ hearing function.

Cross-check principle for the audiometric test battery was maintained. The two audiologists cross-checked each other’s audiological findings. All tested frequencies were repeated twice and correlated with other audiological results obtained. Hearing screening was conducted initially, followed by diagnostic audiometry (see Table 2).

**TABLE 2:** Equipment used and procedures followed in the current study.

Type of assessment	Hearing screening	Diagnostic audiometry
Equipment	Welch-Allyn Otoscope	Welch-Allyn Otoscope
	MT-10 Immittance Meter for tympanometry and ipsilateral reflexes	MT-10 Immittance Meter for tympanometry and ipsilateral reflexes
	Audio-screener for air-conduction pure-tone	Interacoustics AC40 diagnostic audiometer
	Oto-acoustic emissions	Path Medical Solutions – Amtronix Diagnostics: Sentiero Electrophysiology machine
Noise free	16 hours	16 hours
Who conducted the test	Audiologist†	Audiologist†

† , Researcher.

### Ethical consideration

Prior to data collection, the researcher obtained permission from the director of the ASE to conduct the study. Then, individual participants were approached for participation in the study, where verbal consent and written informed consent were obtained from all the participants prior to screening and diagnostic testing. Ethical clearance was obtained from the University Ethics Committee at the University of the Witwatersrand (Non-Medical Ethics Number: STA_2016_11). The study adhered to all ethical considerations set out by the Declaration of Helsinki, revised in 2013 (The Helsinki Declaration of the World Medical Association [WMA] 2014).

### Data analysis

Data obtained were captured on Microsoft Excel spreadsheet and were later represented into frequency tables and graphs. One-sample *t*-test was used to establish the relationship between age and an increased intensity (higher decibels) in the high frequencies. *P*-value was calculated at 95% for this finding in order to confirm the statistical significance.

## Results

Responses for this section were obtained from the self-developed and administered case history questionnaire, hearing screening and diagnostic audiology findings.

**FIGURE 1:**
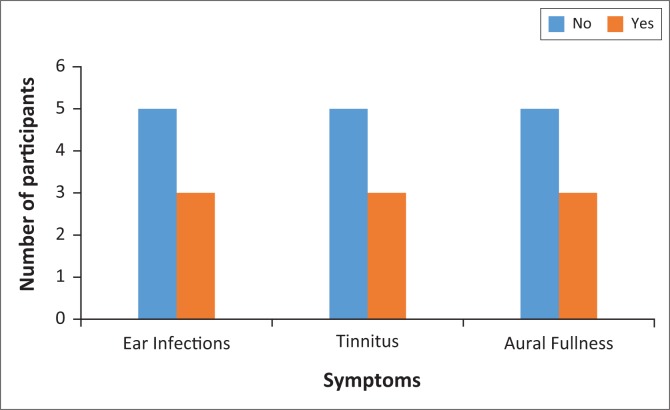
Study participants’ symptomatology.

The case history questionnaire provided data on the participants’ reported symptoms associated with hearing loss. Figure 1 illustrates the participants’ case history information associated with hearing loss. Three participants reported a history of middle ear infections. Of those three, two participants reported recurrent middle ear infections during childhood, and one participant reported to have experienced a last time middle ear infection in 2011 which was treated with antibiotics.

Three participants reported a continuous high-pitched tinnitus throughout the day and night, especially following a work shift where they were exposed to loud music or sounds. There were five participants who stated they did not experience continuous tinnitus, but rather reported only experiencing tinnitus following a live event, which faded when resting the ears in a quiet environment. Three participants reported experiencing a fluctuating aural fullness following a prolonged exposure to loud sounds, but said that aural fullness disappeared the next morning.

### Participants’ previous hearing history

Five participants reported that they had hearing screening conducted before joining ASE. Three participants reported that they have never had their ears tested before. The participants (*N* = 8) reported that they have never undergone diagnostic audiological assessment before.

### Participants’ recreational noise exposure history

**FIGURE 2:**
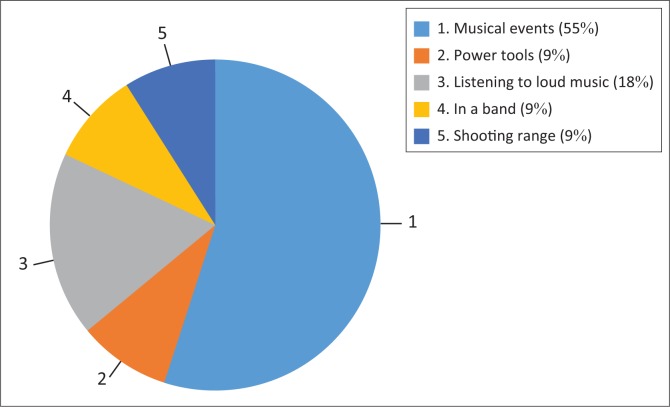
Recreational noise exposure.

Seven participants reported that they were involved in the types of hobbies as listed in Figure 2. Six participants stated that they often attended musical events such as live concerts and music festivals during their spare time. One participant reported that he enjoyed extremely loud music.

### Participants’ hearing function

The results presented in this section were participants’ hearing function that was determined from hearing screening and diagnostic audiometry findings.

#### Hearing screening results

Table 3 illustrates the findings obtained from all eight participants, all presented with hearing within normal limits bilaterally. During otoscopic examination, half (*n* = 4) of the participants presented with impacted cerumen and a dull tympanic membrane. Abnormal middle ear pressure and compliance were obtained from six participants.

**TABLE 3:** Hearing screening results (***N*** = 8).

Test	Equipment	Test findings	Left ear (*n*)	Right ear (*n*)
Otoscopic examination	Otoscope (Welch Allyn)	Impacted cerumen	4	0
		Dull tympanic membrane	1	2
Tympanometry	MT10 (Inter-acoustics)	Pass	4	6
		Refer	4	2
Acoustic reflexes	MT10 (Inter-acoustics)	Pass	5	5
		Refer	3	3
DP oto-acoustic emissions	Path Sentiero	Pass	8	8
Pure tone testing	GSI Audiometer	Pass	8	8

DP, distortion product; GSI, Grason-Stadler Inc.

A ‘pass’ result in pure-tone hearing screening was obtained for all the participants (Roeser, Valente, & Hosford-Dunn, 2007). Distortion product oto-acoustic emission results indicated a ‘pass’ result at all the frequencies tested, bilaterally, for all the participants.

#### Diagnostic audiometry results

**FIGURE 3:**
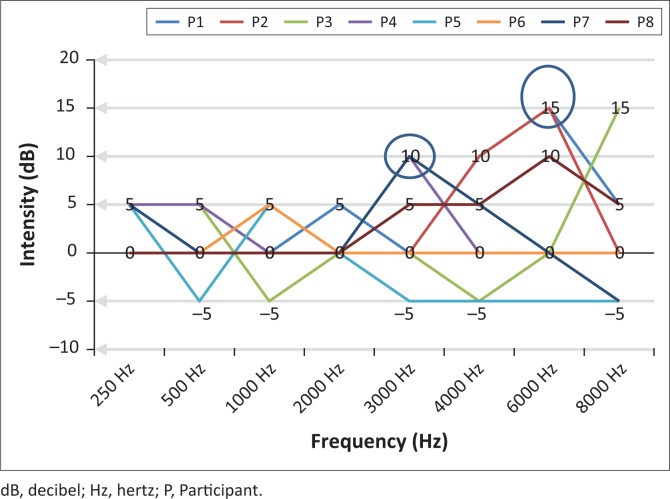
Configuration of participants’ audiograms.

Table 4 and Figure 3 show diagnostic audiometry findings for all the participants. Impacted cerumen was observed in one participant, and all the participants presented with normal otoscopic examination results as well as normal middle ear pressure and compliance. Three participants presented with absent acoustic reflexes bilaterally. Diagnostic audiometry findings from pure-tone audiometry correlated with speech audiometry and indicated hearing within normal limits bilaterally for all the participants. Lastly, oto-acoustic emissions were present for all the participants.

**TABLE 4:** Results from diagnostic audiological testing (*N* = 16 ears)

Test	Test findings	Variable	Left ear (n = 8 ears)					Right ear (*n* = 8 ears)				
			*n*	Ipsilateral		Contralateral		*n*	Ipsilateral		Contralateral	
				Present (70-100dB)	Absent	Present (70-100dB)	Absent		Present (70-100dB)	Absent	Present (70-100dB)	Absent
Otoscopy	NAD*	-	7	-	-	-	-	8	-	-	-	-
	Wax occlusion	-	1	-	-	-	-	0	-	-	-	-
Tymps.	Type A	-	8	-	-	-	-	8	-	-	-	-
Acoustic reflexes	500Hz	-	-	7	1	5	3	-	8	0	7	1
	1000Hz	-	-	6	2	4	4	-	7	1	7	1
	2000Hz	-	-	7	1	4	4	-	7	1	7	1
	4000Hz	-	-	6	2	3	5	-	7	1	7	1
Pure-tone	WNL	-	8	-	-	-	-	8	-	-	-	-
Speech Audio	Speech Reception Threshold (SRT)	Good SRT:PTA < 5dB	8	-	-	-	-	8	-	-	-	-
	Most Comfortable Level (MCL)	WNL (40 dB – 55dB above SRT)	8	-	-	-	-	8	-	-	-	-
	Threshold of Discomfort (TD)	WNL (90 dB – 110dB)	3	-	-	-	-	4	-	-	-	-
		< 90dB	5	-	-	-	-	4	-	-	-	-
	Dynamic Range (DR)	< 60dB	0	-	-	-	-	0	-	-	-	-
		> 60dB	8	-	-	-	-	8	-	-	-	-
	Speech Discrimination	Good discrimination (90% – 100%)	8	-	-	-	-	8	-	-	-	-

Hz, hertz; NAD, no abnormalities detected; Tymps, Tympanometry; PTA, Pure Tone Average; SRT, Speech Reception Threshold; WNL, Within Normal Limits.

Figure 3 illustrates pure-tone audiometry findings for all the participants (*N* = 8). Only three participants (P1, P5 and P6) did not present with a notch of ≥10 dB, in the region between 3000 Hz and 6000 Hz frequency. Five participants’ audiograms indicated a notched configuration in the high frequencies from 3000 Hz (*n* = 2) and 6000 Hz (*n* = 3), with the greatest decrease in hearing thresholds obtained at 15 dB (6000 Hz). The percentage loss of hearing (PLH) was calculated bilaterally using 500 Hz, 1000 Hz, 2000 Hz, 3000 Hz and 4000 Hz frequencies. All eight participants obtained 1.1% PLH value.

For the five participants with increased thresholds at 3000 Hz and 6000 Hz, a *t*-value of 17.6 and *p*-value of 0.001 inferred that at a mean age of 27.6 years (SD = 3.85), there was evidence that at high frequencies of 3000 Hz – 6000 Hz, an increased intensity (notch) would be obtained 95% of the time for these participants.

### Hearing conservation programme

The participants only reported the use of hearing protection devices (HPDs) for hearing loss prevention. Six participants reported wearing HPDs every time they were working at live concerts. The other two participants reported that they avoided wearing earplugs consistently, as this limited their range of hearing music whilst at work. The issue of cost was raised by one participant who indicated that the replacement cost of earplugs was expensive. Disposable earplugs were reported as the most common type of HPDs used.

Results from the study indicated that only four participants knew about NIHL. They knew about the effects of noise on the auditory system and they had been educated on the use of HPDs as a preventative measure for NIHL.

## Discussion

The South African sound engineering population is small and dominated by white men. Our study sample was small but had more white men (*n* = 6). Our sample was also small when compared to the overall sound engineer population in South Africa, which is estimated to be about 4000 nationally (PayScale, 2019). The sample size was small because of the tight teaching schedule of the participants. With one woman participating in the study, this was representative of the South African sound engineering population, where women constitute 8% of the population (PayScale, 2019). The sample had more (76%) mid-career participants, with 3–10 years of experience (PayScale, 2019). Overall, the demographics of our study sample were similar to that of the South African sound engineers according to information presented in the PayScale (2019) website. Although the study sample was representative of the sound engineer demographics, the results may not be generalised to the greater South African sound engineering population.

Tinnitus and aural fullness are common symptoms associated with NIHL. Only three participants in the current study reported these symptoms. In clinical audiology practice, questions about tinnitus and aural fullness are asked in order to establish their presence and impact on one’s hearing function. In a study by Drake-Lee (1992) on rock musicians, the author reported that following a heavy metal concert, participants reported symptoms of temporary blockage (aural fullness) and tinnitus which disappeared the next morning. That study’s findings were similar to the current study findings. Although this study was conducted a while back, common symptoms, tinnitus and aural fullness were common in this population. Tinnitus and aural fullness have been identified as the commonly reported symptoms associated with hearing loss for the sound engineers in our study.

Recreational noise exposure increases one’s susceptibility to NIHL. Activities such as attending live concerts, being in a band and shooting reported by the study participants have been found to be associated with an increased risk of NIHL (Berg, Pickett, Linneman, Wood, & Marlenga, 2014; Cohen, 2015). Data obtained for the current study were subjective because of the lack of actual noise exposure levels, but highlighted confounding factors that increased the participants’ risk to hearing loss. Hearing loss has negative implications for sound engineers, as this affects their ability to produce music (Einhorn, 2012). Therefore, hearing monitoring programmes are imperative for sound engineers in order to prevent any type of hearing loss.

Ear and hearing health promotion emphasises a normal functioning of outer and middle ear. Only one participant had impacted cerumen, and all the participants presented with clear outer ear structures. Unilateral absent acoustic reflexes (ipsilateral and contralateral) obtained, without middle ear pathologies noted in some of our participants, raise concerns with regard to the effectiveness of their stapedius muscle’s protective function. Specific to the study sample, this could be because of excessive levels of music exposure (Wojtczak, Beim, & Oxenham, 2017). Although no studies have reported this finding on the sound engineers, our findings indicated that this (unilateral absence of acoustic reflexes) may be an early sign of hearing loss for this group of participants.

Hearing within normal limits with a notch in the region of 3000 Hz – 6000 Hz is an early sign of NIHL (Rabinowitz, 2000). In a study conducted on sound technicians, the authors (El Dib, Silva, Morais, & Trevisani, 2008) reported a high frequency hearing loss in sound technicians, predominantly in the 25–35 years old age group, which was similar to our findings. In another study conducted by Jansen, Helleman, Dreschler and De Laat (2009), the authors looked at musicians of symphony orchestras using an extensive audiological test battery and found that most musicians could be classified as hearing within normal limits, but their audiograms revealed notches at 6000 Hz. These results correlated with our study findings that indicated that a notch at 6000 Hz was an associated early sign of NIHL, with a notch evident for musicians. Although these studies were conducted more than 5 years apart, their findings were similar and still relevant to our study sample.

Prevention of NIHL requires an effective HCP for sound engineers. The study site did not have an HCP in place, and the participants did not have audiometry records in line with HCP guidelines. Within an HCP, there are seven pillars considered: noise measurement, engineering and administrative controls, education and training, use of HPDs, risk-based medical examination and finally medical surveillance and audiometry (SANS, 2013). None of the pillars existed at our study site. Without an HCP in place, concerns are raised with regard to the company’s intention of hearing loss prevention strategy for this study site. Professional sound engineers and sound engineering students need to be aware of noise and the consequent effects of noise exposure on hearing, and the importance of hearing loss prevention within the HCP framework (SANS, 2013).

## Conclusion and recommendations

The main aim of this study was to determine the hearing function of sound engineers and to establish if HCPs were in place at the sound engineers’ academy in Johannesburg, South Africa. In conclusion, all the participants presented with hearing within normal limits bilaterally. However, participants’ results revealed the following early signs of NIHL: reported tinnitus and aural fullness, unilateral absent acoustic reflexes (ipsilateral and contralateral), and a notch at 3000 Hz and 6000 Hz frequencies obtained from pure-tone results (*n* = 5). Without an HCP set at the academy, participants could continue to lose their hearing gradually, leading to NIHL. This study raised awareness on the importance of creating a sound engineering-specific HCP that would benefit professionals at the study site.

### Limitations

The limitations of this study are the following:

- A sample size of eight participants was too small for the findings to be generalised.
- The participants were full-time lecturers and not full-time sound engineers. Therefore, their experiences were supposedly different from that of full-time sound engineers.
- Participants were not randomly selected.
- Noise exposure levels were not measured; therefore, there was uncertainty whether levels exceeded 85 dBA over 8 hours.
